# Reliability of Muscle Strength and Muscle Power Assessments Using Isokinetic Dynamometry in Neuromuscular Diseases: A Systematic Review

**DOI:** 10.1093/ptj/pzac099

**Published:** 2022-07-28

**Authors:** Danny R van der Woude, Thijs Ruyten, Bart Bartels

**Affiliations:** Child Development and Exercise Center, Wilhelmina Children’s Hospital, University Medical Center Utrecht, Utrecht, the Netherlands; Child Development and Exercise Center, Wilhelmina Children’s Hospital, University Medical Center Utrecht, Utrecht, the Netherlands; Child Development and Exercise Center, Wilhelmina Children’s Hospital, University Medical Center Utrecht, Utrecht, the Netherlands

**Keywords:** Muscle Strength, Muscle Strength Dynamometer, Muscle Weakness, Neuromuscular Diseases, Reproducibility of Results

## Abstract

**Objective:**

The purpose of this study was to critically appraise and summarize the evidence for reliability of muscle strength and muscle power assessment in patients with neuromuscular diseases (NMDs) using isokinetic dynamometry.

**Methods:**

PubMed, CINAHL, and Embase electronic databases were searched from inception to March 8, 2022. Studies designed to evaluate reliability of muscle strength and power measurements using isokinetic dynamometry were included in this review. First, the methodological quality of the studies was assessed according to the Consensus-Based Standards for the Selection of Health Measurement Instruments guidelines. Next, the quality of measurement properties was determined. Finally, the methodological quality and quality of measurement properties of the studies were combined to obtain a best-evidence synthesis.

**Results:**

A best-evidence synthesis of reliability was performed in 11 studies including postpoliomyelitis syndrome (n = 5), hereditary motor and sensory neuropathy (n = 2), motor neuron diseases (n = 1), myotonic dystrophy (n = 1), and groups of pooled NMDs (n = 2). A best-evidence synthesis on measurement error could not be performed. Quality of evidence on reliability ranged from high in postpoliomyelitis syndrome to very low in hereditary motor and sensory neuropathy, motor neuron diseases, and groups of pooled NMDs. The most frequently used outcome measure was peak torque, which was reliable in all populations (intraclass correlation coefficient >0.7).

**Conclusion:**

The quality of evidence for reliability of isokinetic dynamometry was found to vary substantially among different NMDs. High quality of evidence has been obtained only in patients with postpoliomyelitis syndrome. Further research is needed in the majority of known NMDs to determine reliability and validity of isokinetic dynamometry.

**Impact:**

The ability of isokinetic dynamometers to capture clinically relevant changes in muscle strength and muscle power in NMDs remains unclear. Isokinetic dynamometry results in NMDs should be interpreted with caution.

## Introduction

Neuromuscular disorders (NMDs) are a heterogeneous group of acquired and genetic diseases that may encompass all levels of dysfunction of the peripheral nervous system—that is, the anterior horn cells, the peripheral nerve, and the neuromuscular junction—as well as muscle itself.^1^ The large majority of NMDs are characterized by muscle weakness.^1–3^ Muscle function assessments are generally used to determine disease severity, gain insight into disease trajectory, and assess therapeutic efficacy.^4^^,^^5^ To meet these objectives, reliable and sensitive outcome measures for muscle strength and muscle power are required.^6^

Various methods are available to assess muscle function. Manual muscle testing and handheld dynamometry (HHD) are most commonly used for quantitative strength measurements due to their low cost and ease of use.^7^ Both measures provide insight into the maximum strength in a fixed position (ie, isometric strength).^8^ Manual muscle testing uses a 6-point manual scale (the Medical Research Council scale) to evaluate muscle strength ranging from grade 0 (no contraction noticeable) to grade 5 (complete range of motion against gravity and hold the test position against maximum resistance).^9^ HHD registers the force one can produce against a portable handheld dynamometer in a fixed position and allows quantification in newtons.^10^ Despite the broad and longstanding application of manual muscle testing and HHD in clinical practice, both techniques have limitations; manual muscle testing lacks sensitivity to differentiate between moderate weakness to normal strength,^6^^,^^11^^,^^12^ whereas HHD reliability is limited in stronger muscle groups.^7^ In studies in which ceiling effects may come into play, alternative techniques such as isokinetic dynamometry might represent better options.

Isokinetic dynamometry is considered the “gold standard” of muscle strength and muscle power measurements in orthopedic and neurological patients.^13–16^ Isokinetic dynamometers (IDs) have superior sensitivity and have higher reliability compared with HHD testing in adults who are healthy.^13^ IDs are designed to quantify both isometric and isokinetic strength.^17^ Isometric strength measurements are performed with the limb in a fixed position measuring the muscle’s ability to develop static force, measured as torque in newton-meters. Isokinetic strength is defined as the ability of a muscle to develop dynamic force. Power (watts) refers to the ability to produce force over a specified period of time and range.^8^ Isokinetic muscle strength and muscle power are more reflective of daily life activities than isometric measurements.^18^^,^^19^

To properly assess the added value of isokinetic dynamometry in the evaluation of muscle function in patients with NMDs, the reliability of the method needs to be assessed.^20^ According to the Consensus-Based Standards for the Selection of Health Measurement Instruments (COSMIN) guidelines, reliability refers to the degree to which measurements are free from error. The domain of reliability is subdivided into the measurement properties of reliability and measurement error.^21^ The proportion of the total variance of the measurement due to true differences is defined as reliability, expressed in Intraclass Correlation Coefficients (ICCs) or Pearson *r.*^21^^,^^22^ In addition to the reliability of results, it is important to know which part of the difference between repeated measurements is attributed to systematic and random error, the measurement error.^21^^,^^23^ The variability between repeated measurements can be expressed in different standards, including the standard error of measurement (SEM), limits of agreement, and the smallest detectable change.^22^^,^^23^

Several studies have explored the reliability of strength and power measurements using IDs in patients with NMD, but no systematic review to our knowledge has been performed on the methodological quality of measurement properties and applicability of isokinetic dynamometry. Here, we conducted a literature review to determine the current level of evidence for reliability of isometric and isokinetic muscle strength and muscle power assessment in patients with NMDs using isokinetic dynamometers.

## Methods

### Data Sources and Searches

We searched relevant articles in the electronic databases of PubMed, CINAHL, and Embase up to March 8, 2022. Reference lists of included articles were additionally searched for relevant studies. The search was not constrained by either timespan or language. Depending on the search engine MESH terms, Emtree words and keywords were implemented in the search string. We used the search filter as described by Terwee et al^24^ to optimize the yield of relevant articles. The full search strategy is listed in Supplementary Appendix 1.

**Figure:**
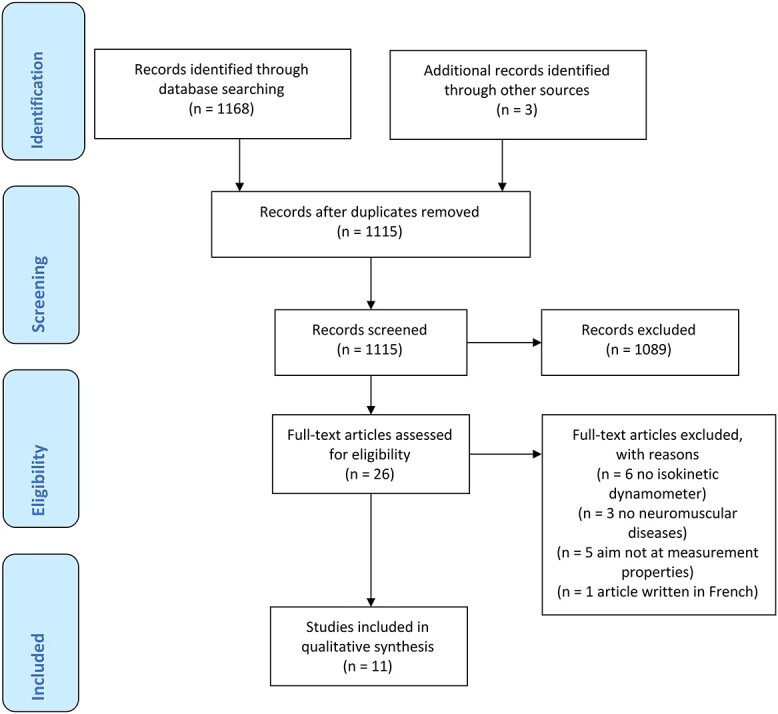
Study selection.

### Study Selection

One author (D.R.W.) performed the database search. Subsequently, 2 reviewers (D.R.W. and T.R.) independently selected articles that met the inclusion criteria. Articles were eligible for inclusion when the following 4 criteria were met: the aim of the article was to describe measurement properties of strength and/or power measurements using an isokinetic dynamometer; the articles described reliability and/or measurement error; the population tested included individuals with neuromuscular disease(s); and articles were written in either the English or Dutch language. The 3 exclusion criteria were as follows: no full-text article was available, the particular dynamometer used was incompletely specified, and isokinetic dynamometric measurements were used to validate other tests or evaluate interventions.

We screened all articles on title and abstract. Articles potentially eligible for the study were attained in full text and further examined for inclusion. Articles selected by both reviewers were included. Articles selected by only 1 reviewer were discussed until consensus was reached. If needed, a third reviewer (B.B.) was consulted.

### Data Extraction and Quality Assessment

#### Data Extraction

Descriptive data and data on measurement properties were extracted from each study by 1 author (D.R.W.). Three steps were taken after data extraction, based on the COSMIN guidelines.^21^ First, we assessed the methodological quality of the studies. Second, we determined the quality of measurement properties. Finally, we combined both methodological quality and quality measurement properties of the studies in a best-evidence synthesis.

#### Quality Assessment

Two reviewers (D.R.W. and T.R.) independently assessed the methodological quality of the included studies following the COSMIN risk of bias checklist.^25^ This is a standardized tool to assess the methodological quality of studies on measurement properties. The risk of bias checklist contains 10 measurement properties that can be scored on a 4-point scale.^25^

We rated the methodological quality of the relevant measurement properties (ie, reliability and measurement error) as very good, adequate, doubtful, inadequate, or not applicable. The lowest rating of any standard in the box was then used according to the “worst score counts” principle.^21^ In case of disagreement regarding the methodological quality, the 2 principal reviewers discussed the paper in detail until consensus was reached. If not, a third reviewer (B.B.) was consulted.

The quality of the measurement properties was rated sufficient, indeterminate, or insufficient based on criteria provided by the COSMIN guidelines (Tab. 1).

**Table 1:** Quality Criteria for Good Measurement Properties*^a^*

**Measurement Property**	**Rating**	**Criteria**
Reliability	Sufficient	ICC or weighted κ ≥ 0.70
	Indeterminate	ICC or weighted κ not reported
	Insufficient	ICC or weighted κ < 0.70
Measurement error	Sufficient	SDC or LoA < minimal important change
	Indeterminate	Minimal important change not defined
	Insufficient	SDC or LoA > minimal important change

^a^ LoA = limits of agreement; SDC = smallest detectable change.

### Data Synthesis and Analysis

#### Quality of Evidence

To summarize the quality of evidence, we performed a best-evidence synthesis based on methodological quality and quality of measurement properties. The level of evidence was described per diagnosis, type of measurement (ie, isometric or isokinetic), and outcome measure (ie, peak torque, power, angle at peak, maximum isometric torque). We graded the quality of evidence as high, moderate, low, or very low based on the Grading of Recommendations Assessment, Development and Evaluation approach.^21^ Starting with the assumption of high-quality evidence, scores were downgraded for the factors risk of bias, inconsistency, imprecision, and indirectness.

## Results

### Included Studies

We identified a total of 11 articles for this study (the flowchart is shown in the Figure). The kappa agreement between the reviewers in selecting articles after application of the inclusion and exclusion criteria was 0.92.

### Study Characteristics

The topics of the included 11 articles were postpoliomyelitis syndrome (5 articles),^26–30^ hereditary motor and sensory neuropathy (HMSN) (2 articles),^31^^,^^32^ myotonic dystrophy type 1 (1 article),^33^ and motor neuron disease (1 article)^34^; 2 studies contained information on a group with more than 1 NMD^35^^,^^36^ (Tab. 2). All studies enrolled adult patients ranging in age from 24 to 77 years. Types of dynamometers used were Biodex (multi-joint) System 3 (Pro),^26–28^^,^^33^^,^^35^ Biodex System 4 Pro,^33^ Cybex 2,^29^^,^^32^ Cybex 6000,^36^ Kin-Com,^30^ Kin-Com 2,^34^ and the Lido Active Multi-Joint 2.^31^ All studies reported details of testing position and fixation used. Three studies reported the number of patients unable to perform all measurement procedures because of muscle weakness.^26^^,^^29^^,^^31^

**Table 2:** Study Characteristics and Test Procedures*^a^*

**Study**	**Diagnosis**	**No. of Participants (Men/Women)**	**Age, y**		**Dynamometer**	**Time Between Test and Retest**	**Measurement**				
							**Isometric**		**Isokinetic**		
			**Mean (SD)**	**Range**			**Movement**	**Outcome**	**Speed**	**Movement**	**Outcome**
Andersen^31^ (1996)	HMSN	7 (3/4)	46	24–70	Lido Active Multi-Joint 2	At least 24 h			30, 60, 180°/s	Ankle dorsal flexion-plantar flexion	Peak torque
Brogårdh et al^26^ (2015)	Postpoliomyelitis syndrome	28 (16/12)	68	29–81	Biodex System 3 Pro	14 d	Shoulder abduction, elbow flexion	Highest MVC	60°/s	Elbow flexion-extension	Peak torque
Fillyaw et al^32^ (1989)	HMSN	15 (10/5)	48.3 (9.6)		Cybex 2 isokinetic dynamometer	1–2 mo	Shoulder flexion- extension, elbow flexion- extension, knee flexion- extension, ankle dorsal flexion-plantarflexion	Maximum isometric torque			
Flansbjer and Lexell^28^ (2010)	Postpoliomyelitis syndrome	30 (19/11)	63	51–77	Biodex Multi-Joint System 3 Pro	7 d	Knee extension	Highest MVC	60°/s	Knee flexion-extension	Peak torque
Flansbjer et al^27^ (2011)	Postpoliomyelitis syndrome	31*^b^* (18/13)	63	50–77	Biodex Multi-Joint System 3 Pro	7 d	Ankle dorsal flexion	Highest MVC	30°/s	Ankle dorsal flexion	Peak torque
Griffin et al^34^ (1994)	Motor neuron diseases	11*^c^* (NA)	NA		Kin-Com 2	1 wk			30°,120°/s	Isokinetic knee flexion-extension	Average torque
Horemans et al^30^ (2004)	Postpoliomyelitis syndrome	65 (23/42)	52	36–68	Kin-Com	3 wk	Knee extension	Highest MVC			
Kilfoil and St Pierre^29^ (1993)	Postpoliomyelitis syndrome	8*^d^* (4/4)	Men: 47.5 (10); women: 56.8 (16)		Cybex 2	1–3 wk			1.05,2.09, 3.14,4.18 rads∙s^−1^	Knee flexion-extension	Peak torque, angle at peak torque, maximum power, average power
Knak et al^33^ (2020)	Noncongenital myotonic dystrophy	78 (39/39)	40 (10)		Biodex System 3 Pro and System 4 Pro	1–2 wk	Knee flexion- extension, hip flexion- extension, ankle plantar flexion, and dorsal flexion	MVC			
Tiffreau et al^36^ (2003)	Multiple NMDs pooled	14 (7/7)	Men: 35.2 (8.4); women: 38.7 (11.2)		Cybex 6000	7 d			10°/s	Knee flexion-extension (continuous passive movement mode)	Mean work, maximum work
Tiffreau et al^35^ (2007)	Multiple NMDs pooled	15 (3/12)	40.4	16–67	Biodex 3 isokinetic dynamometer	At least 2 h			10°,30°/s	Knee flexion-extension (continuous passive movement mode)	Peak torque, angle at peak torque, work, power

^a^ HMSN = hereditary motor and sensory neuropathy; MVC = maximum voluntary contraction (ie, peak torque); NA = not available; NMDs = neuromuscular diseases.

^b^ Seventeen individuals were unable to perform the measurement with the more affected limb because of muscle weakness.

^c^ Five patients with motor neuron diseases were included in the reliability assessment.

^d^ Three patients were not eligible for bilateral evaluation.

Eight studies evaluated the reliability of isokinetic measurements; 5 of these studies tested knee flexion-extension,^28^^,^^29^^,^^35^^,^^36^ 2 tested ankle plantar flexion and dorsal flexion,^27^^,^^31^ and 1 tested elbow flexion and extension.^26^ Six studies reported the reliability of isometric measurements: 4 for knee-flexion and/or extension,^28^^,^^30^^,^^32^^,^^33^ 3 for ankle plantar flexion and dorsal flexion,^27^^,^^32^^,^^33^ 2 for elbow flexion,^26^^,^^32^ and 2 for shoulder abduction.^26^^,^^32^

A total of 6 of 8 studies used peak torque in isokinetic measurements. The highest maximum voluntary contraction (MVC) or maximum isometric torque (ie, peak torque) was used in all 6 studies that contained isometric measurements.

Six studies evaluated measurement error; 4 of these studies reported on knee flexion and/or extension,^28^^,^^30^^,^^32^^,^^33^ 3 reported on ankle dorsal flexion and plantar flexion,^27^^,^^32^^,^^33^ and 2 reported on elbow flexion, elbow extension, and shoulder abduction.^26^^,^^29^ A summary of all measurement properties is presented in Supplementary Appendix 2.

#### Methodological Quality

The methodological quality of the 11 selected articles is presented in Table 3. Eight studies demonstrated adequate quality,^26–30^^,^^33^^,^^35^^,^^36^ whereas 3 studies showed doubtful methodological quality on reliability.^31^^,^^32^^,^^34^ Five studies demonstrated an adequate methodological quality on measurement error,^26–28^^,^^30^^,^^33^ whereas 1 study was considered to have a doubtful methodological quality on measurement error.^32^ The main methodological flaws included the lack of information on patients being stable during the test–retest period, although it could be assumed in most studies. Three studies lacked sufficiently detailed description of the statistics used,^29^^,^^31^^,^^32^ which affected both reliability and measurement error scores. Supplementary Appendix 3. shows details of COSMIN scores on each criterion.

**Table 3:** Methodological Quality*^a^*

**Diagnosis**	**Study**	**Reliability**	**Measurement Error**
Postpoliomyelitis syndrome	Brogårdh et al^26^ (2015)	Adequate	Adequate
	Flansbjer and Lexell^28^ (2010)	Adequate	Adequate
	Flansbjer et al^27^ (2011)	Adequate	Adequate
	Horemans et al^30^ (2004)	Adequate	Adequate
	Kilfoil and St Pierre^29^ (1993)	Adequate	Indeterminate
HMSN	Andersen^31^ (1996)	Doubtful	Indeterminate
	Fillyaw et al^32^ (1989)	Doubtful	Doubtful
Motor neuron diseases	Griffin et al^34^ (1994)	Doubtful	Indeterminate
Myotonic dystrophy type 1	Knak et al^33^ (2020)	Adequate	Adequate
Pooled NMDs	Tiffreau et al^36^ (2003)	Adequate	Indeterminate
	Tiffreau et al^35^ (2007)	Adequate	Indeterminate

^a^ HMSN = hereditary motor and sensory neuropathy; NMDs = neuromuscular diseases.

#### Quality of Measurement Properties

Eight studies reported on the reliability of isokinetic measurements^26–29^^,^^31^^,^^34–36^ (Tab. 4). All outcomes but angle at peak torque showed sufficient reliability (ICC = 0.7–0.99) on various speeds, joints and movements in postpoliomyelitis syndrome, HMSN, motor neuron diseases, and combinations of NMDs. The reliability of isometric measurements was determined in 6 studies.^26–28^^,^^30^^,^^32^^,^^33^ The highest MVC was sufficiently reliable in postpoliomyelitis syndrome (ICC = 0.92–0.98). In myotonic dystrophy type 1, torque had a sufficiently reliable outcome measure (ICC = 0.79–0.97). In HMSN, maximum isometric torque was sufficiently reliable in shoulder and elbow movements (ICC = 0.84–0.997).

**Table 4:** Synthesis of Results for Reliability*^a^*

**Diagnosis**	**Type of Measurement**	**Total No. of Participants**	**Outcome Used**	**ICC Range**	**Quality of Measurement Properties: Reliability**	**Risk of Bias**	**Inconsistency**	**Imprecision**	**Indirectness**	**Quality of Evidence**
Postpoliomyelitis syndrome	Isometric	154	Highest MVC	0.92–0.98	Sufficient	0	0	0	0	High
	Isokinetic	97	Highest peak torque	0.81–0.99	Sufficient	0	0	−1	0	Moderate
		8	Angle at peak torque	0.20–0.91	Insufficient	−1	0	−2	0	Very low
		8	Average power	0.88–0.98	Sufficient	−1	0	−2	0	Very low
		8	Maximum power	0.84–0.98	Sufficient	−1	0	−2	0	Very low
HMSN	Isometric	30	Maximum isometric torque	0.84–0.96	Sufficient	−2	0	−2	0	Very low
	Isokinetic	4–7	Highest peak torque	0.975–0.997*^b^*	Sufficient	−2	0	−2	0	Very low
Myotonic dystrophy type 1	Isometric	58–72	Torque	0.79–0.97	Sufficient	−1	0	−1	0	Low
Motor neuron diseases	Isokinetic	5	Average torque	≥0.97	Sufficient	−2	0	−2	0	Very low
Multiple pooled NMDs	Isokinetic	15	Highest peak torque	0.92–0.99	Sufficient	−1	0	−2	0	Very low
		15	Angle at peak torque	0.56–0.90	Insufficient	−1	0	−2	0	Very low
		15	Maximum power	0.94–0.98	Sufficient	−1	0	−2	0	Very low
		14	Mean work	0.78–0.84	Sufficient	−1	0	−2	0	Very low
		14	Maximum work	0.7–0.83	Sufficient	−1	0	−2	0	Very low

^a^ HMSN = hereditary motor and sensory neuropathy; MVC = maximum voluntary contraction; NMDs = neuromuscular diseases.

^b^ *r* values.

Six studies reported on measurement error^26–28^^,^^30^^,^^32^^,^^33^; 3 of these studies reported the smallest detectable change. Of these, 2 studies investigated postpoliomyelitis and 1 study investigated myotonic dystrophy type 1.^26^^,^^28^^,^^33^ The quality of the measurement properties for measurement error were indeterminate due to the absence of reported anchor-based minimal important change.^21^ Additional data on measurement properties can be found in Supplementary Appendix 2.

### Quality of Evidence

Evidence of high quality was found for isometric measurements of strength in postpoliomyelitis syndrome using the highest MVC as outcome measure. Isokinetic measurements in postpoliomyelitis syndrome showed only moderate quality of evidence when highest peak torque was used as the outcome measure. For other NMDs, quality of evidence was low to very low for both isometric and isokinetic measurements (Tab. 4) as a result of small sample sizes and doubtful methodological quality.

#### Reliability

Evidence of high quality was found for isometric measurements of strength in postpoliomyelitis syndrome using the highest MVC as outcome measure. Isokinetic measurements in postpoliomyelitis syndrome showed only moderate quality of evidence when highest peak torque was used as the outcome measure. For other NMDs, quality of evidence was low to very low for both isometric and isokinetic measurements (Tab. 4) as a result of small sample sizes and doubtful methodological quality.

#### Measurement Error

We could not perform a best-evidence synthesis of measurement error because the quality of the measurement property could not be determined.

## Discussion

We found moderate to high evidence that measuring strength and power using an isokinetic dynamometer in postpoliomyelitis syndrome is reliable. For other NMDs, the quality of evidence on reliability was low to very low, mostly because of small sample sizes and doubtful methodological quality of the included studies. The quality of evidence for measurement error was indefinable for all NMDs because of the absence of reported anchor-based minimal clinically important differences.

In terms of reliability, the findings of this systematic review are similar to the results of reviews on adults who were healthy^13^ and for isokinetic knee strength measures in children.^37^ They are comparable with results of reliability studies in patients with fibromyalgia,^38^ heart failure,^39^ and chronic obstructive pulmonary disease,^40^ which all concluded that IDs are reliable tools for measuring strength and/or power. A previous study by Mhandi et al^41^ focusing on the use of IDs in NMDs summarized the reliability of isokinetic measurements in NMDs as high based solely on ICCs. The present study shows comparable results on ICCs and additionally provides the quality of evidence in different groups of NMDs.

Measurement errors of strength testing in NMDs are similar to studies of adults who were healthy for isokinetic measurements of knee (SEM = 5.4–17.3 N·m) and ankle (SEM = 0.9–5.7 N·m) and isometric knee strength (SEM = 6.2–8.1 N·m).^13^ Compared with adults who were healthy, patients with NMDs showed comparable to slightly higher measurement error, expressed as SEM%, which indicates the relative measurement error (SEM divided by the group mean × 100). SEM% is comparable in isometric knee measurements (6.4%–13.0% in NMDs vs 3.6%–15.9% in adults who were healthy) and slightly higher in isometric hip measurements (14.0%–15.0% in NMDs vs 6.5%–10.8% in adults who were healthy) and ankle measurements (12.4%–30.0% in NMDs vs 5.8%–13.8% in adults who were healthy).^13^ Higher SEM% compared with adults who were healthy may reflect the overall lower strength in patients with NMDs given that absolute SEMs are comparable.^13^

Multiple studies have showed the impact of force conductor placement, rest before and between measurements, and use of the handrail on strength and power measured. With respect to the former, Andersen reported that a 3-cm displacement of the force conductor impacted the outcome of plantar flexor strength measurement by as much as 23 N·m.^31^ Rest has been shown to impact strength measurement outcomes in both adults who were healthy and patients with NMDs.^31^^,^^42^ Nunes et al reported differences in strength outcome and reliability while using the handrails in isokinetic knee strength measurements.^43^ These findings demonstrate the importance of meticulous description of any protocol used to assess muscle function using isokinetic dynamometry.

Three studies included in our search outcome used the continuous passive mode (CPM) on 7 patients with HMSN^31^ and 2 groups with multiple NMDs and with strength ranging from Medical Research Council grade 2 to Medical Research Council grade 5.^31^^,^^35^^,^^36^ In isokinetic measurements using CPM, the dynamometer moves at a constant speed and supports the tested limb through a preset range of motion. CPM is described as a valid mode to measure torque, making it possible to measure strength in patients with Medical Research Council grades as low as 2 and 3.^44^^,^^45^ The included studies that used CPM showed similar reliability compared with the other studies, indicating IDs might not only be of use in stronger but also in weaker patients.

The described test procedures per study can be used to create reliable strength measurements, and the reported SEM allows to determine whether improvement and decrease in strength are true changes.

Clinicians can use the outcome of this study to set up a protocol for strength and power measurements in NMDs when using isokinetic dynamometers. The synthesis of results provided by the present investigation summarizes the current quality of evidence for the use of isokinetic dynamometers in measuring strength and/or power in NMDs.

### Limitations

#### Limitations of the Systematic Review

This study used the COSMIN criteria to include studies. The COSMIN was developed to measure measurement properties of patient-reported outcomes. Several reviews show that the guideline can also be used to review studies on clinical outcome measures.^46–48^

The COSMIN guidelines only include studies with measurement properties as a specific research aim. Studies focusing on other specific aims such as validation of HHD that additionally reported on reliability were excluded.^49^ This review did not address feasibility. Although feasibility is not part of reliability, it affects the clinical applicability of isokinetic dynamometry. In this review, we compared outcomes obtained with different IDs, although a recent study did not show differences between the Biodex System 3 Pro and the Cybex Humac Norm 770. However, it is not enough evidence that there is no difference at all between IDs. Therefore, we cannot rule out the influence of differences between IDs.^50^ We therefore recommend the use of the same ID for specific patient groups.

#### Limitations of the Reviewed Studies

Multiple studies^26–29^^,^^31^ excluded patients who did not reach the preset velocity in isokinetic measurements. Thus, reliability and measurement error are determined for a subgroup of the population and do not represent the entire population. Only 2 studies included more than 50 patients, which may be considered an adequate number of patients to determine reliability estimates.^51^ With limited samples sizes and only a few studies at each NMD, only the highest MVC measured isometrically in post-poliomyelitis patients was not scored down for imprecision for having <100 participants.

One study used the Pearson *r* to measure reliability in isokinetic measurements in HMSN. Although the COSMIN guidelines score down on the methodological quality of the study, a correlation >0.7 is considered sufficient when scoring the measurement properties. Comparison with ICCs can be misleading even when ICCs and *r* point in the same direction of high correlation/reliability.

This systematic review provides an overview of strength and power testing in NMDs using isokinetic dynamometers. We found that the quality of evidence regarding the reliability of measuring strength and power with isokinetic dynamometers in neuromuscular diseases was variable between NMDs. Specifically, isometric measurements in postpoliomyelitis syndrome were found to have high reliability, whereas reliability was low to very low for isometric and isokinetic measurements in HMSN and other NMDs. This review has identified highest peak torque or highest MVC as the most commonly used and reliable outcome, whereas angle at peak torque should be considered an inappropriate outcome measure.

### Recommendations

Studies with larger samples sizes are needed to create reliable protocols and useful reliability data for measuring patients with NMDs. An accurate description of the protocol used can improve reliability. To improve reliability, the authors recommend using the arms crossed on the chest for knee measurements and to standardize patient and force conductor placement. Highest peak torque/highest MVC show the highest quality of evidence and highest reliability and are therefore recommended as an outcome measure. For weaker patients, the use of lower velocities in isokinetic testing or measurements using CPM is recommended. In stronger patients, higher velocities are recommended because they have a higher correlation with walking. Finally, the authors recommend the use of standardized rest intervals between measurements to limit the effect of fatigue.

Further research on the use of CPM in isokinetic measurements is useful for the measurement of power in patients with lower strength who are unable to achieve the preset velocity.

## Supplementary Material

Supplementary_Appendix_1_pzac099

Supplementary_Appendix_2_pzac099

Supplementary_Appendix_3_pzac099

## Systematic Review Registration

This review was not registered.
