# Supplementary material for: Reliability of Muscle Strength and Muscle Power Assessments Using Isokinetic Dynamometry in Neuromuscular Diseases: A Systematic Review
Source: Phys Ther. 2022 Jul 28;102(10):pzac099. doi: 10.1093/ptj/pzac099 (PMC10071497; doi:10.1093/ptj/pzac099)
Supplement: Supplementary_Appendix_1_pzac099 [file supplementary_appendix_1_pzac099.pdf]

Appendix 1

search string

PUBMED

((((("Neuromuscular Diseases"[MeSH Terms] OR"muscle disease*"[Title/Abstract] OR ("neuromuscular junction*"[Title/Abstract] OR ("neuromuscular disease*"[Title/Abstract] OR "motor neuron disease*"[Title/Abstract])))))

AND

((((((((("isometric*"[Title/Abstract] OR "isokinetic*"[Title/Abstract]) OR "Muscle Strength Dynamometer"[MeSH Terms]) OR "biodex*"[Title/Abstract]) OR "IMT"[Title/Abstract]) OR "Torque"[MeSH Terms]) OR "muscle fatigue*"[Title/Abstract]))))) OR cybex[Title/Abstract]))

AND

((("instrumentation"[MeSH Subheading] OR "reproducibility of results"[MeSH Terms]) OR "reproducib*"[Title/Abstract]) OR "psychometrics"[MeSH Terms]) OR "psychometr*"[Title/Abstract]) OR "clinimetr*"[Title/Abstract]) OR "clinometr*"[Title/Abstract]) OR "observer variation"[MeSH Terms]) OR "observer variation"[Title/Abstract]) OR "discriminant analysis"[MeSH Terms]) OR "reliab*"[Title/Abstract]) OR "valid*"[Title/Abstract]) OR "coefficient"[Title/Abstract]) OR "internal consistency"[Title/Abstract]) OR ("cronbach*"[Title/Abstract] AND

("alpha"[Title/Abstract] OR "alphas"[Title/Abstract]))) OR "item correlation"[Title/Abstract]) OR "item correlations"[Title/Abstract]) OR "item selection"[Title/Abstract]) OR "item selections"[Title/Abstract]) OR "item reduction"[Title/Abstract]) OR "item reductions"[Title/Abstract]) OR "agreement"[Text Word]) OR "precision"[Text Word]) OR "imprecision"[Text Word]) OR "precise values"[Text Word]) OR "test-retest"[Title/Abstract]) OR ("test"[Title/Abstract] AND "retest"[Title/Abstract])) OR ("reliab*"[Title/Abstract] AND ("test"[Title/Abstract] OR "retest"[Title/Abstract]))) OR "stability"[Title/Abstract]) OR "interrater"[Title/Abstract]) OR "inter-rater"[Title/Abstract]) OR "intrarater"[Title/Abstract]) OR "intra-rater"[Title/Abstract]) OR "intertester"[Title/Abstract]) OR "inter-tester"[Title/Abstract]) OR "intratester"[Title/Abstract]) OR "intra-tester"[Title/Abstract]) OR "interobserver"[Title/Abstract]) OR "inter-observer"[Title/Abstract]) OR "intraobserver"[Title/Abstract]) OR "intra-observer"[Title/Abstract]) OR "intertechnician"[Title/Abstract]) OR "inter-technician"[Title/Abstract]) OR "intratechnician"[Title/Abstract]) OR "intra-technician"[Title/Abstract]) OR "interexaminer"[Title/Abstract]) OR "inter-examiner"[Title/Abstract]) OR "intraexaminer"[Title/Abstract]) OR "intra-examiner"[Title/Abstract]) OR "interassay"[Title/Abstract]) OR "inter-assay"[Title/Abstract]) OR "intraassay"[Title/Abstract]) OR "intra-assay"[Title/Abstract]) OR "interindividual"[Title/Abstract]) OR "inter-individual"[Title/Abstract]) OR "intraindividual"[Title/Abstract]) OR "intra-individual"[Title/Abstract]) OR "interparticipant"[Title/Abstract]) OR "inter-participant"[Title/Abstract]) OR "intraparticipant"[Title/Abstract]) OR "intra-participant"[Title/Abstract]) OR "kappa"[Title/Abstract]) OR "kappa's"[Title/Abstract]) OR "kappas"[Title/Abstract]) OR "coefficient of variation"[Title/Abstract]) OR "repeatab*"[Text Word]) OR (("replicab*"[Text Word] OR "repeated"[Text Word]) AND (((((("measure"[Text

Word] OR "measures"[Text Word]) OR "findings"[Text Word]) OR "result"[Text Word]) OR "results"[Text Word]) OR "test"[Text Word]) OR "tests"[Text Word]))) OR "generaliza*"[Title/Abstract]) OR "generalisa*"[Title/Abstract]) OR "concordance"[Title/Abstract]) OR ("intraclass"[Title/Abstract] AND "correlation*"[Title/Abstract])) OR "discriminative"[Title/Abstract]) OR "known group"[Title/Abstract]) OR "factor analysis"[Title/Abstract]) OR "factor analyses"[Title/Abstract]) OR "factor structure"[Title/Abstract]) OR "factor structures"[Title/Abstract]) OR "dimensionality"[Title/Abstract]) OR "subscale*"[Title/Abstract]) OR "multitrait scaling analysis"[Title/Abstract]) OR "multitrait scaling analyses"[Title/Abstract]) OR "item discriminant"[Title/Abstract]) OR "interscale correlation"[Title/Abstract]) OR "interscale correlations"[Title/Abstract]) OR (("error"[Title/Abstract] OR "errors"[Title/Abstract]) AND (((((("measure*"[Title/Abstract] OR "correlat*"[Title/Abstract]) OR "evaluat*"[Title/Abstract]) OR "accuracy"[Title/Abstract]) OR "accurate"[Title/Abstract]) OR "precision"[Title/Abstract]) OR "mean"[Title/Abstract]))) OR "individual variability"[Title/Abstract]) OR "interval variability"[Title/Abstract]) OR "rate variability"[Title/Abstract]) OR "variability analysis"[Title/Abstract]) OR ("uncertainty"[Title/Abstract] AND ("measurement"[Title/Abstract] OR "measuring"[Title/Abstract]))) OR "standard error of measurement"[Title/Abstract]) OR "sensitiv*"[Title/Abstract]) OR "responsive*"[Title/Abstract]) OR ("limit"[Title/Abstract] AND "detection"[Title/Abstract])) OR "minimal detectable concentration"[Title/Abstract]) OR "interpretab*"[Title/Abstract]) OR (("small*"[Title/Abstract] AND ("real"[Title/Abstract] OR "detectable"[Title/Abstract])) AND ("change"[Title/Abstract] OR "difference"[Title/Abstract]))) OR "meaningful change"[Title/Abstract]) OR "minimal important change"[Title/Abstract]) OR "minimal important difference"[Title/Abstract]) OR

"minimally important change"[Title/Abstract]) OR "minimally important difference"[Title/Abstract]) OR "minimal detectable change"[Title/Abstract]) OR "minimal detectable difference"[Title/Abstract]) OR "minimally detectable change"[Title/Abstract]) OR "minimally detectabl e difference"[Title/Abstract]) OR ((((((((((((("minimal"[All Fields] OR "minimisation"[All Fields]) OR "minimisations"[All Fields]) OR "minimise"[All Fields]) OR "minimised"[All Fields]) OR "minimises"[All Fields]) OR "minimising"[All Fields]) OR "minimization"[All Fields]) OR "minimizations"[All Fields]) OR "minimize"[All Fields]) OR "minimized"[All Fields]) OR "minimizer"[All Fields]) OR "minimizers"[All Fields]) OR "minimizes"[All Fields]) OR "minimizing"[All Fields]) AND "real change"[Title/Abstract])) OR "minimal real difference"[Title/Abstract]) OR ("minimally"[All Fields] AND "real change"[Title/Abstract])) OR ("minimally"[All Fields] AND "real difference"[Title/Abstract])) OR "ceiling effect"[Title/Abstract]) OR "floor effect"[Title/Abstract]) OR "item response model"[Title/Abstract]) OR "IRT"[Title/Abstract]) OR "Rasch"[Title/Abstract]) OR "differential item functioning"[Title/Abstract]) OR "DIF"[Title/Abstract]) OR "computer adaptive testing"[Title/Abstract]) OR "item bank"[Title/Abstract]) OR "cross cultural equivalence"[Title/Abstract])))))

NOT

((((((((((((((("biography"[Publication Type] OR "case reports"[Publication Type]) OR "comment"[Publication Type]) OR "directory"[Publication Type]) OR "editorial"[Publication Type]) OR "festschrift"[Publication Type]) OR "interview"[Publication Type]) OR "legislation"[Publication Type]) OR "letter"[Publication Type]) OR "news"[Publication Type]) OR "newspaper article"[Publication Type]) OR "patient education handout"[Publication Type])

OR "consensus development conference"[Publication Type]) OR "consensus development conference, nih"[Publication Type]) OR "practice guideline"[Publication Type])) NOT ("animals"[MeSH Terms] NOT "humans"[MeSH Terms])))

EMBASE

(((reproducib*:ti,ab OR 'psychometry'/exp OR 'psychometry' OR psychometr*:ti,ab OR clinimetr*:ti,ab OR clinometr*:ti,ab OR 'observer'/exp OR observer) AND ('variation'/exp OR 'variation') OR 'observer variation':ti,ab OR discriminant) AND ('analysis'/exp OR 'analysis') OR reliab*:ti,ab OR valid*:ti,ab OR coefficient:ti,ab OR 'internal consistency':ti,ab OR (cronbach*:ti,ab AND (alpha:ti,ab OR alphas:ti,ab)) OR 'item correlation':ti,ab OR 'item correlations':ti,ab OR 'item selection':ti,ab OR 'item selections':ti,ab OR 'item reduction':ti,ab OR 'item reductions':ti,ab OR 'agreement[text word]' OR 'precision[text word]' OR 'imprecision[text word]' OR 'precise values[text word]' OR 'test retest':ti,ab OR (test:ti,ab AND retest:ti,ab) OR (reliab*:ti,ab AND (test:ti,ab OR retest:ti,ab)) OR stability:ti,ab OR interrater:ti,ab OR 'inter rater':ti,ab OR intrarater:ti,ab OR 'intra rater':ti,ab OR intertester:ti,ab OR 'inter tester':ti,ab OR intratester:ti,ab OR 'intra tester':ti,ab OR interobserver:ti,ab OR 'inter observer':ti,ab OR intraobserver:ti,ab OR 'intra observer':ti,ab OR intertechnician:ti,ab OR 'inter technician':ti,ab OR intratechnician:ti,ab OR 'intra technician':ti,ab OR interexaminer:ti,ab OR 'inter examiner':ti,ab OR intraexaminer:ti,ab OR 'intra examiner':ti,ab OR interassay:ti,ab OR 'inter assay':ti,ab OR intraassay:ti,ab OR 'intra assay':ti,ab OR interindividual:ti,ab OR 'inter individual':ti,ab OR intraindividual:ti,ab OR 'intra individual':ti,ab OR interparticipant:ti,ab OR 'inter participant':ti,ab OR intraparticipant:ti,ab OR 'intra participant':ti,ab OR kappa:ti,ab OR kappas:ti,ab OR

'coefficient of variation':ti,ab OR 'repeatab*[text word]' OR (('replicab*[text word]' OR 'repeated[text word]') AND ('measure[text word]' OR 'measures[text word]' OR 'findings[text word]' OR 'result[text word]' OR 'results[text word]' OR 'test[text word]' OR 'tests[text word]')) OR generaliza*:ti,ab OR generalisa*:ti,ab OR concordance:ti,ab OR (intraclass:ti,ab AND correlation*:ti,ab) OR discriminative:ti,ab OR 'known group':ti,ab OR 'factor analysis':ti,ab OR 'factor analyses':ti,ab OR 'factor structure':ti,ab OR 'factor structures':ti,ab OR dimensionality:ti,ab OR subscale*:ti,ab OR 'multitrait scaling analysis':ti,ab OR 'multitrait scaling analyses':ti,ab OR 'item discriminant':ti,ab OR 'interscale correlation':ti,ab OR 'interscale correlations':ti,ab OR ((error:ti,ab OR errors:ti,ab) AND (measure*:ti,ab OR correlat*:ti,ab OR evaluat*:ti,ab OR accuracy:ti,ab OR accurate:ti,ab OR precision:ti,ab OR mean:ti,ab)) OR 'individual variability':ti,ab OR 'interval variability':ti,ab OR 'rate variability':ti,ab OR 'variability analysis':ti,ab OR (uncertainty:ti,ab AND (measurement:ti,ab OR measuring:ti,ab)) OR 'standard error of measurement':ti,ab OR sensitiv*:ti,ab OR responsive*:ti,ab OR (limit:ti,ab AND detection:ti,ab) OR 'minimal detectable concentration':ti,ab OR interpretab*:ti,ab OR (small*:ti,ab AND (real:ti,ab OR detectable:ti,ab) AND (change:ti,ab OR difference:ti,ab)) OR 'meaningful change':ti,ab OR 'minimal important change':ti,ab OR 'minimal important difference':ti,ab OR 'minimally important change':ti,ab OR 'minimally important difference':ti,ab OR 'minimal detectable change':ti,ab OR 'minimal detectable difference':ti,ab OR 'minimally detectable change':ti,ab OR 'minimally detectabl e difference':ti,ab)

AND

('neuromuscular diseases'/exp OR 'neuromuscular diseases' OR 'muscle disease*':ti,ab OR 'neuromuscular junction*':ti,ab OR 'neuromuscular disease*':ti,ab OR 'motor neuron disease*':ti,ab)

AND

(isometric*:ti,ab OR isokinetic*:ti,ab OR 'dynamometer'/exp OR 'dynamometer' OR biodex*:ti,ab OR imt:ti,ab OR 'torque'/exp OR 'torque' OR 'muscle fatigue*':ti,ab OR cybex:ti,ab)

CINAHL

((((((MH "Neuromuscular Diseases+") OR TI "muscle disease*" OR AB "muscle disease*" OR (TI "neuromuscular junction*" OR AB "neuromuscular junction*" OR (TI "neuromuscular disease*" OR AB "neuromuscular disease*" OR TI "motor neuron disease*" OR AB "motor neuron disease*")))))

AND

(((((((((TI isometric* OR AB isometric* OR TI isokinetic* OR AB isokinetic*) OR (MH "Muscle Strength Dynamometer+")) OR TI biodex* OR AB biodex*) OR TI IMT OR AB IMT) OR (MH "Torque+")) OR TI "muscle fatigue*" OR AB "muscle fatigue*"))))) OR TI cybex OR AB cybex))

AND

((
"instrumentation[MeSH Subheading]" OR (MH "reproducibility of results+")) OR TI reproducib* OR AB reproducib*) OR (MH "psychometrics+")) OR TI psychometr* OR AB psychometr*) OR TI clinimetr* OR AB clinimetr*) OR TI clinometr* OR AB clinometr*) OR (MH "observer variation+")) OR TI "observer variation" OR AB "observer variation") OR (MH "discriminant analysis+")) OR TI reliab* OR AB reliab*) OR TI valid* OR AB valid*) OR TI coefficient OR AB coefficient) OR TI "internal consistency" OR AB "internal consistency") OR (TI cronbach* OR AB cronbach* AND (TI alpha OR AB alpha OR TI alphas OR AB alphas))) OR TI "item correlation" OR AB "item correlation") OR TI "item correlations" OR AB "item correlations") OR TI "item selection" OR AB "item selection") OR TI "item selections" OR AB "item selections") OR TI "item reduction" OR AB "item reduction") OR TI "item reductions" OR AB "item reductions") OR "agreement[Text Word]") OR "precision[Text Word]") OR "imprecision[Text Word]") OR "precise values[Text Word]") OR TI test-retest OR AB test-retest) OR (TI test OR AB test AND TI retest OR AB retest)) OR (TI reliab* OR AB reliab* AND (TI test OR AB test OR TI retest OR AB retest))) OR TI stability OR AB stability) OR TI interrater OR AB interrater) OR TI inter-rater OR AB inter-rater) OR TI intrarater OR AB intrarater) OR TI intra-rater OR AB intra-rater) OR TI intertester OR AB intertester) OR TI inter-tester OR AB inter-tester) OR TI intratester OR AB intratester) OR TI intra-tester OR AB intra-tester) OR TI interobserver OR AB interobserver) OR TI inter-observer OR AB inter-observer) OR TI intraobserver OR AB intraobserver) OR TI intra-observer OR AB intra-observer) OR TI intertechnician OR AB intertechnician) OR TI inter-technician OR AB inter-technician) OR TI intratechnician OR AB intratechnician) OR TI intra-technician OR AB intra-technician) OR TI interexaminer OR AB interexaminer) OR TI inter-examiner OR AB inter-examiner) OR TI

intraexaminer OR AB intraexaminer) OR TI intra-examiner OR AB intra-examiner) OR TI interassay OR AB interassay) OR TI inter-assay OR AB inter-assay) OR TI intraassay OR AB intraassay) OR TI intra-assay OR AB intra-assay) OR TI interindividual OR AB interindividual) OR TI inter-individual OR AB inter-individual) OR TI intraindividual OR AB intraindividual) OR TI intra-individual OR AB intra-individual) OR TI interparticipant OR AB interparticipant) OR TI inter-participant OR AB inter-participant) OR TI intraparticipant OR AB intraparticipant) OR TI intra-participant OR AB intra-participant) OR TI kappa OR AB kappa) OR TI kappa's OR AB kappa's) OR TI kappas OR AB kappas) OR TI "coefficient of variation" OR AB "coefficient of variation") OR "repeatab*[Text Word]") OR (("replicab*[Text Word]" OR "repeated[Text Word]") AND (((((("measure[Text Word]" OR "measures[Text Word]") OR "findings[Text Word]") OR "result[Text Word]") OR "results[Text Word]") OR "test[Text Word]") OR "tests[Text Word]"))) OR TI generaliza* OR AB generaliza*) OR TI generalisa* OR AB generalisa*) OR TI concordance OR AB concordance) OR (TI intraclass OR AB intraclass AND TI correlation* OR AB correlation*)) OR TI discriminative OR AB discriminative) OR TI "known group" OR AB "known group") OR TI "factor analysis" OR AB "factor analysis") OR TI "factor analyses" OR AB "factor analyses") OR TI "factor structure" OR AB "factor structure") OR TI "factor structures" OR AB "factor structures") OR TI dimensionality OR AB dimensionality) OR TI subscale* OR AB subscale*) OR TI "multitrait scaling analysis" OR AB "multitrait scaling analysis") OR TI "multitrait scaling analyses" OR AB "multitrait scaling analyses") OR TI "item discriminant" OR AB "item discriminant") OR TI "interscale correlation" OR AB "interscale correlation") OR TI "interscale correlations" OR AB "interscale correlations") OR ((TI error OR AB error OR TI errors OR AB errors) AND ((((((TI measure* OR AB measure* OR TI correlat* OR AB correlat*) OR TI evaluat* OR AB evaluat*) OR TI accuracy OR AB accuracy) OR TI accurate OR AB accurate) OR TI precision OR AB precision) OR TI mean OR AB mean)))

OR TI "individual variability" OR AB "individual variability") OR TI "interval variability" OR AB "interval variability") OR TI "rate variability" OR AB "rate variability") OR TI "variability analysis" OR AB "variability analysis") OR (TI uncertainty OR AB uncertainty AND (TI measurement OR AB measurement OR TI measuring OR AB measuring))) OR TI "standard error of measurement" OR AB "standard error of measurement") OR TI sensitiv* OR AB sensitiv*) OR TI responsive* OR AB responsive*) OR (TI limit OR AB limit AND TI detection OR AB detection)) OR TI "minimal detectable concentration" OR AB "minimal detectable concentration") OR TI interpretab* OR AB interpretab*) OR ((TI small* OR AB small* AND (TI real OR AB real OR TI detectable OR AB detectable)) AND (TI change OR AB change OR TI difference OR AB difference))) OR TI "meaningful change" OR AB "meaningful change") OR TI "minimal important change" OR AB "minimal important change") OR TI "minimal important difference" OR AB "minimal important difference") OR TI "minimally important change" OR AB "minimally important change") OR TI "minimally important difference" OR AB "minimally important difference") OR TI "minimal detectable change" OR AB "minimal detectable change") OR TI "minimal detectable difference" OR AB "minimal detectable difference") OR TI "minimally detectable change" OR AB "minimally detectable change") OR TI "minimally detectabl e difference" OR AB "minimally detectabl e difference") OR (((((((((((((((minimal OR minimisation) OR minimisations) OR minimise) OR minimised) OR minimises) OR minimising) OR minimization) OR minimizations) OR minimize) OR minimized) OR minimizer) OR minimizers) OR minimizes) OR minimizing) AND TI "real change" OR AB "real change")) OR TI "minimal real difference" OR AB "minimal real difference") OR (minimally AND TI "real change" OR AB "real change")) OR (minimally AND TI "real difference" OR AB "real difference")) OR TI "ceiling effect" OR AB "ceiling effect") OR TI "floor effect" OR AB "floor effect") OR TI "item response model" OR AB "item response model") OR

TI IRT OR AB IRT) OR TI Rasch OR AB Rasch) OR TI "differential item functioning" OR AB "differential item functioning") OR TI DIF OR AB DIF) OR TI "computer adaptive testing" OR AB "computer adaptive testing") OR TI "item bank" OR AB "item bank") OR TI "cross cultural equivalence" OR AB "cross cultural equivalence")))

NOT

(((((((((((((((("biography[Publication Type]" OR "case reports[Publication Type]") OR "comment[Publication Type]") OR "directory[Publication Type]") OR "editorial[Publication Type]") OR "festschrift[Publication Type]") OR "interview[Publication Type]") OR "legislation[Publication Type]") OR "letter[Publication Type]") OR "news[Publication Type]") OR "newspaper article[Publication Type]") OR "patient education handout[Publication Type]") OR "consensus development conference[Publication Type]") OR "consensus development conference, nih[Publication Type]") OR "practice guideline[Publication Type]")) NOT ((MH "animals+") NOT (MH "humans+")))
