# Supplementary material for: Reliability of Muscle Strength and Muscle Power Assessments Using Isokinetic Dynamometry in Neuromuscular Diseases: A Systematic Review
Source: Phys Ther. 2022 Jul 28;102(10):pzac099. doi: 10.1093/ptj/pzac099 (PMC10071497; doi:10.1093/ptj/pzac099)
Supplement: Supplementary_Appendix_2_pzac099 [file supplementary_appendix_2_pzac099.pdf]

Appendix 2

Summary of measurement properties

Study	Type of protocol	Reliability		Measurement error	
Postpoliomyelitis syndrome					
**Brogårdh et al.** **(**2015) SWE	Isokinetic (60°/sec)<br>Highest peak torque	Less affected side	More affected side	Less affected side	More affected side
	Elbow flexion	ICC=.94	ICC=.90	SEM=3.6 (12%)<br>SDC=10.0 (34%)	SEM=4.0 (16%)<br>SDC =11.0 (45%)
	Elbow extension	ICC=.87	ICC=.89	SEM=5.1 (20%)<br>SDC =14.1 (57%)	SEM=4.6 (24%)<br>SDC =12.9 (67%)
	Isometric<br>Highest MVC				
	Shoulder abduction	ICC=.95	ICC=.94	SEM=4.3 (14%)<br>SDC =12.0 (38%)	SEM=4.0 (17%)<br>SDC =11.0 (48%)
	Elbow flexion	ICC=.92	ICC=.96	SEM=5.5 (14%)<br>SDC =15.3(40%)	SEM=3.5 (12%)<br>SDC =9.7 (33%)
**Flansbjer et al.** **(**2010) SWE	Isokinetic (60°/sec) Highest peak torque	Less affected side	More affected side	Less affected side	More affected side
	Knee extension	ICC=.94	ICC=.98	SEM= 11.1 (13.9%)<br>SDC = NA (38.5%)	SEM=6.7 (10.2%)<br>SDC = NA (28.4%)
	Knee flexion	ICC=.93	ICC=.95	SEM=6.8 (10.4%)<br>SDC = NA (28.7%)	SEM=5.1 (11.8%)<br>SDC = NA (32.8%)
	Isometric<br>Highest MVC				
	Knee extension	ICC=.98	ICC=.98	SEM=8.8 (6.4%)<br>SDC = NA (17.8%)	SEM=7.4 (9.0%)<br>SDC = NA (24.9%)
**Flansbjer et al.** **(**2011) SWE	Isokinetic (30°/sec) Highest peak torque	Less affected side	More affected side	Less affected side	More affected side
	Ankle dorsi flexion	ICC=.93	ICC=.85	SEM=2.51 (9.7%)	SEM=3.74 (16.4%)
	Isometric<br>Highest MVC				
	Ankle plantar flexion	ICC=.93	ICC=.94	SEM=3.2 (12.4%)	SEM=3.03 (13%)
**Horemans et al.** (2004) NL	Isometric<br>Highest MVC (peak torque)<br>knee extension	ICC=.96		LoA=.75-1.29	
**Kilfoil et al.** (1993) USA	Isokinetic (1.05 rads·s^{-1})	Stronger limb (in patients in which both limbs are tested)	Weaker limb		
	Knee flexion				
	Peak torque	ICC=.95	ICC=.95		
	Angle peak torque	ICC=.91	ICC=.39		
	Average power	ICC=.97	ICC=.91		
	Maximum power	ICC=.97	ICC=.95		
	Knee extension				
	Peak torque	ICC=.95	ICC=.82		
	Angle peak torque	ICC=.88	ICC=.90		
	Average power	ICC=.93	ICC=.93		
	Maximum power	ICC=.98	ICC=.89		
	Isokinetic (2.09 rads·s^{-1})				
	Knee flexion				
	Peak torque	ICC=.83	ICC=.81		
	Angle peak torque	ICC=.26	ICC=.82		
	Average power	ICC=.97	ICC=.95		
	Maximum power	ICC=.94	ICC=.95		
	Knee extension				
	Peak torque	ICC=.95	ICC=.99		
	Angle peak torque	ICC=.56	ICC=.88		
	Average power	ICC=.94	ICC=.98		
	Maximum power	ICC=.92	ICC=.94		
	Isokinetic (3.14 rads·s^{-1})				
	Knee flexion				
	Peak torque	ICC=.91	ICC=.92		

	Angle peak torque	ICC=.20	ICC=.70	
	Average power	ICC=.96	ICC=.88	
	Maximum power	ICC=.90	ICC=.84	
	Knee extension			
	Peak torque	ICC=.87	ICC=.98	
	Angle peak torque	ICC=.54	ICC=.84	
	Average power	ICC=.98	ICC=.98	
	Maximum power	ICC=.90	ICC=.98	
	Isokinetic (4.18 rads·s^{-1})			
	Knee flexion			
	Peak torque	ICC=.98	ICC=.94	
	Angle peak torque	ICC=.57	ICC=.46	
	Average power	ICC=.98	ICC=.91	
	Maximum power	ICC=.98	ICC=.93	
	Knee extension			
	Peak torque	ICC=.98	ICC=.99	
	Angle peak torque	ICC=.72	ICC=.34	
	Average power	ICC=.97	ICC=.98	
	Maximum power	ICC=.94	ICC=.98	
HMSN				
**Andersen** (1996) DEN	Isokinetic. (30°/sec, Continuous passive movement mode)			
	Peak torque			
	Dorsal flexion	r=.990		
	Plantairflexion	r=.975		
	Isokinetic. (60°/sec)			
	Peak torque			
	Ankle dorsalflexion	r=.987		
	Ankle plantairflexion	r=.997		
**Fillyaw et al.** (1989) USA	Isometric.			
	Maximum isometric torque			
	Shoulder flexion	ICC=.96		SEM=2.97
	Shoulder extension	ICC=.96		SEM=3.74
	Elbow flexion	ICC=.95		SEM=3.02
	Elbow extension	ICC=.94		SEM=2.98
	Knee flexion	ICC=.91		SEM=6.49
	Knee extension	ICC=.89		SEM=14.07
	Ankle dorsalflexion	ICC=.84		SEM=1.12
	Ankle plantarflexion	ICC=.86		SEM=7.43
Myotnic Dystrophy type 1				
**Knak et al.** (2020) DEN				
	Isometric			
	Torque			
	Hip flexion	ICC=.87		SEM=9.56(15%) SDC=26.5 (25%)
	Hip extension	ICC=.92		SEM=1.14(14%) SDC=1.45 (38%)
	Knee flexion	ICC=.91		SEM=7.87 (13%) SDC=21.8 (25%)
	Knee extension	ICC=.96		SEM=1.10(10%) SDC=1.32 (24%)
	Ankle dorsal flexion	ICC=.91		SEM=3.40 (15%) SDC=29.43 (17%)
	Ankle plantar flexion	ICC=.79		SEM=1.30(30%) SDC=2.07 (71%)
Motor neuron diseases				
**Griffin et al.** (1993) USA	Isokinetic knee extension. Eccentric and concentric. 30°/sec and 120°/sec			
	Average torque	ICC≥.97		
	Isokinetic knee flexion. Eccentric and concentric. 30°/sec and 120°/sec			
	Average torque	ICC≥.98		
Pooled NMDs				

**Tiffreau et al.** (2003) FRA	Isokinetic.10°/sec (Continuous passive movement mode)		
	Knee flexion		
	Mean work	ICC=.78	
	Maximal work	ICC=.70	
	Knee extension		
	Mean work	ICC=.84	
	Maximal work	ICC=.83	
**Tiffreau et al. (**2017) FRA	Isokinetic.10°/sec (Continuous passive movement mode)		
	Knee flexion	Right knee	Left knee
	Peak torque	ICC=.95	ICC=.97
	Angle at peak	ICC=.79	ICC=.85
	Power	ICC=.94	ICC=.98
	Knee extension		
	Peak torque	ICC=.92	ICC=.98
	Angle at peak	ICC=.90	ICC=.77
	Power	ICC=.96	ICC=.97
	Isokinetic.30°/sec (Continuous passive movement mode)		
	Knee flexion		
	Peak torque	ICC=.97	ICC=.98
	Angle at peak	ICC=.90	ICC=.56
	Power	ICC=.96	ICC=.99
	Knee extension		
	Peak torque	ICC=.99	ICC=.97
	Angle at peak	ICC=.90	ICC=.76
	Power	ICC=.99	ICC=.95

ICC= intraclass correlation, MVC= maximal voluntary contraction, SEM= standard error of measurement SEM%=relative value of SEM, SRD= Smallest real difference, LoA= Limits of agreement.
