# Supplementary material for: Reliability of Muscle Strength and Muscle Power Assessments Using Isokinetic Dynamometry in Neuromuscular Diseases: A Systematic Review
Source: Phys Ther. 2022 Jul 28;102(10):pzac099. doi: 10.1093/ptj/pzac099 (PMC10071497; doi:10.1093/ptj/pzac099)
Supplement: Supplementary_Appendix_3_pzac099 [file supplementary_appendix_3_pzac099.pdf]

Appendix 3

Cosmin rating of methodological quality

	Reliability								Measurement error	
**Study**	**Were patients stable in the interim period on the construct to be measured?***	**Was the time interval appropriate? ***	**Were the test conditions similar for the measurements? e.g. type of administration, environment, instructions***	**For continuous scores: Was an intraclass correlation coefficient (ICC) calculated?**	**For dichotomous/nominal/ordinal scores: Was kappa calculated?**	**For ordinal scores: Was a weighted kappa calculated?**	**For ordinal scores: Was the weighting scheme described? e.g. linear, quadratic**	**Were there any other important flaws in the design or statistical methods of the study?***	**For continuous scores: Was the Standard Error of Measurement (SEM), Smallest Detectable Change (SDC) or Limits of Agreement 9LoA)**	**For dichotomous/nominal/ordinal scores: Was the percentage (positive and negative) agreement calculated?**
Andersen[31]	Adequate	Very good	Adequate	Doubtful	Not applicable	Not applicable	Not applicable	Very good	Not applicable	Not applicable
Brogårdh et al.[26]	Adequate	Very good	Very good	Very good	Not applicable	Not applicable	Not applicable	Very good	Very good	Not applicable
Fillyaw et al.[32]	Adequate	Adequate/ Doubtful	Doubtful	Adequate	Not applicable	Not applicable	Not applicable	Doubtful	Adequate	Not applicable
Flansbjer et al.[28]	Adequate	Very good	Very good	Very good	Not applicable	Not applicable	Not applicable	Very good	Very good	Not applicable
Flansbjer et al.[27]	Adequate	Very good	Very good	Very good	Not applicable	Not applicable	Not applicable	Very good	Very good	Not applicable
Griffin et al.[34]	Adequate	Adequate	Doubtful	Adequate	Not applicable	Not applicable	Not applicable	Doubtful	Not applicable	Not applicable
Horemans et al.[30]	Adequate	Very good	Adequate	Very good	Not applicable	Not applicable	Not applicable	Very good	Very good	Not applicable
Kilfoil et al.[29]	Adequate	Very good	Adequate	Adequate	Not applicable	Not applicable	Not applicable	Very good	Not applicable	Not applicable
Knak et al.[33]	Adequate	Very good	Adequate	Very good	Not applicable	Not applicable	Not applicable	Very good	Very good	Not applicable
Tiffreau et al.[36]	Adequate	Adequate	Very good	Very good	Not applicable	Not applicable	Not applicable	Very good	Not applicable	Not applicable
Tiffreau et al.[35]	Adequate	Very good	Adequate	Adequate	Not applicable	Not applicable	Not applicable	Very good	Not applicable	Not applicable

*Applies for methodological quality of both reliability and measurement error.
